# Influence of Culture Medium Composition and Light Conditions on the Accumulation of Bioactive Compounds in Shoot Cultures of *Scutellaria lateriflora* L. (American Skullcap) Grown In Vitro

**DOI:** 10.1007/s12010-017-2508-2

**Published:** 2017-06-01

**Authors:** Beata Kawka, Inga Kwiecień, Halina Ekiert

**Affiliations:** Department of Pharmaceutical Botany, Jagiellonian University, Collegium Medicum, Medyczna Street 9, 30-688 Kraków, Poland

**Keywords:** American skullcap, Secondary metabolites, Baicalin, Verbascoside, Flavonoids, Plant growth regulators, Darkness, Monochromatic light

## Abstract

Methanolic extracts from in vitro grown *Scutellaria lateriflora* shoots cultured on five Murashige and Skoog (MS) medium variants supplemented with different combinations of 6-benzylaminopurine (BAP) and α-naphthaleneacetic acid (NAA) under different light conditions (monochromatic light, white light and no light) were analysed by HPLC for three groups of metabolites: flavonoids (26 compounds), phenolic acids and their precursors (19+2) and phenylethanoid glycosides (2). The analyses revealed the presence of baicalein, baicalin, wogonin, wogonoside, 3,4-dihydroxyphenylacetic acid and verbascoside. There was clear evidence of the influence of plant growth regulators and light conditions on the accumulation of the analysed groups of secondary metabolites. The amounts of the compounds changed within a wide range—for the total flavonoid content, 30.2-fold (max. 1204.3 mg·100 g^−1^ dry weight (DW)); for 3,4-dihydroxyphenylacetic acid, 5.5-fold (max. 33.56 mg·100 g^−1^ DW); and for verbascoside, 1.5-fold (169.15 max. mg·100 g^−1^ DW). The best medium for the production of most of the compounds was the Murashige and Skoog variant with 1 mg l^−1^ BAP and 1 mg l^−1^ NAA. For verbascoside, the best ‘productive’ medium was the MS variant supplemented with 0.5 mg l^−1^ BAP and 2 mg l^−1^ NAA. The accumulation of the metabolites was stimulated to the greatest extent by blue light, under which the extracts were found to contain the highest total amount of flavonoids and the highest amounts of flavonoid glucuronides, baicalin and wogonoside, as well as of verbascoside. Their amounts were, respectively, 1.54-, 1.49-, 2.05- and 1.86-fold higher than under the control white light.

## Introduction

*Scutellaria lateriflora* L. (American skullcap) has its natural habitats in wetland areas of North America [1]. It is grown on a large scale in the USA as well as in Chile, Mexico and Costa Rica. This species is obtained only occasionally from its natural habitats because of its similarity to *Teucrium canadense* (American germander) and *Teucrium chamaedrys* (Wall germander). The infusion of the aerial parts of this species has been used by the indigenous peoples of North America for centuries as a diuretic, anticonvulsant and sedative [2]. The raw materials of *S. lateriflora* L. are the aerial parts of the plant, which currently have a monograph in the American Herbal Pharmacopoeia [3]. In modern medicine, this raw material is used as an anxiolytic, a sedative and tonic for the nervous system [4]. It is recommended for the treatment of insomnia and also for treating addiction to drugs and alcohol [2]. *S. lateriflora* can also be used in the emergency treatment of chronic gastrointestinal smooth muscle spasms and as a detoxicant, which is also useful in the case of the withdrawal syndrome. The pharmacological action of the raw material is due to the presence of flavonoids specific for the genus *Scutellaria*, mainly glycoside conjugates of baicalein and wogonin, baicalin and wogonoside [5, 6]. These compounds have potent antiinflammatory, antibacterial [7], antioxidant [8], hepatoprotective and anticancer effects, among others [9]. In addition to the specific flavonoids, the aerial parts also contain flavonoids that are popular in the world of plants, such as apigenin and luteolin [10]. Other components of significance include iridoids, essential oil, resin and tannins and small amounts of phenolic acids. From the group of phenolic acids, analyses have confirmed the presence of caffeic, *p*-coumaric and ferulic acids and of a precursor of one of the subgroups of phenolic acids–cinnamic acid [11]. The raw material has also been found to contain a phenylethanoid glycoside–verbascoside [12]. This compound has a very strong antioxidant action and shows a DNA-protective activity. It also has an antineurodegenerative effect, which is important for the treatment of Parkinson’s disease [13]. Verbascoside is also known for its antibacterial and antiviral properties [14].

The latest phytochemical studies of the aerial parts of *S. lateriflora* L. have confirmed the presence of baicalin and wogonoside as the leading flavonoids. Using various methods of extraction and detection, the following compounds have been identified: baicalin, baicalein, chrysin, dihydrobaicalin, lateriflorin, oroxylin A, oroxylin A 7-*O*-glucuronide, scutellarin, wogonin and wogonoside, and also daidzein, gankwanin, hesperetin, hesperidin, ikonnikoside I, naringenin, quercetin and rutin [15, 16]. Also confirmed is the presence of GABA, glutamine and trace amounts of the amino acids: tryptophan, phenylalanine, proline, glutamic acid, arginine, asparagine, aspartic acid, tyrosine, isoleucine, leucine and valine [4, 17].

The amounts of flavonoids specific to the leaves of *S. lateriflora* can reach more than 5% in the plant raw material. However, numerous analyses of commercially available herbal preparations, including the results of our own research, do not confirm such high quantities of these compounds. This is evidence of considerable diversity in the population of this species, as well as of the impact of the conditions and duration of storage of the raw material on its chemical composition.

Biotechnological studies of the species have mainly been focused around the possibility of using cultures of hairy roots as a source of secondary metabolites. They have been concerned with the effects of the conditions of culturing, the presence and absence of light and elicitation (methyl jasmonate, cyclodextrins, yeast extract and lysates of bacterial strains) [12, 18]. In hairy root cultures, the largest accumulated amounts were those of baicalin and wogonoside [18] and also of verbascoside and scutellarin [12]. Those quantities were lower than in hairy root cultures of another medicinal species of the genus *Scutellaria*—e.g. of the Baikal skullcap (*S. baicalensis*). Cultures of *S. lateriflora* hairy roots have also been found to contain four phenolic acids (salicylic, ferulic, sinapic and vanillic) [4].

Studies of untransformed *S. lateriflora* cultures in vitro have demonstrated the presence of mainly baicalin and wogonin in shoot cultures, whereas the predominant metabolite in callus tissue was verbascoside (2.5%), accompanied by trace amounts of flavonoids [19]. The available literature also contains information on the micropropagation of this species [20, 21].

External factors such as nutrient levels, especially the type and concentration of growth regulators, as well as light conditions (wavelength, intensity, photoperiod, including absence of light) have a marked effect on the accumulation of secondary metabolites in plants cultured under in vitro conditions [22–24].

The aim of this study was to determine the effects of 6-benzylaminopurine (BAP) in combination with α-naphthaleneacetic acid (NAA), in a concentration range of 0.5–3 mg l^−1^, and different light conditions (darkness and monochromatic light: red, blue, UV-A), with white light as control conditions, on the accumulation of flavonoids and other phenolic compounds in stationary shoot cultures of *S. lateriflora* L. maintained on Murashige–Skoog (MS) media variants [25]. The study included both flavonoids specific to the genus *Scutellaria* and flavonoid compounds popular in the world of plants. In addition, the research on phenolic compounds included analyses of phenolic acids and phenylethanoid glycosides, verbascoside and isoverbascoside. This is the first comprehensive study of the biosynthetic potential of *S. lateriflora* in vitro cultures testing of the effects of the concentration of growth regulators in culture media and the effects of illumination conditions on the accumulation of secondary metabolites. These results may help to propose this valuable American species into European phytotherapy.

## Experimental

### Establishment of In Vitro Cultures

In vitro shoot cultures of *S. lateriflora* L. were derived from shoot parts (above the cotyledons) of 4-week-old seedlings grown at 24 ± 2 °C on the Murashige–Skoog [25] initiating medium containing 3% (*w*/*v*) sucrose, 0.7% (*w*/*v*) phytoagar (Duchefa Biochemie, Netherlands) as gelling agent, with the addition of 2 mg l^−1^ BAP and 2 mg l^−1^ NAA. The medium was adjusted to pH 5.7 before autoclaving. The seeds used to produce the seedlings were acquired in 2015 from W. J. Beal Botanical Garden, Michigan State University (USA).

### Experimental Cultures

Shoot cultures were maintained on five different variants (four vials per variant, 1 g of explants each) of MS medium containing different concentrations of plant growth regulators: variant MSI (1 mg l^−1^ BAP and 1 mg l^−1^ NAA), variant MSII (2 mg l^−1^ BAP and 2 mg l^−1^ NAA), variant MSIII (3 mg l^−1^ BAP and 1 mg l^−1^ NAA), variant MSIV (0.5 mg l^−1^ BAP and 2 mg l^−1^ NAA) and variant MSV (1 mg l^−1^ BAP and 0.5 mg l^−1^ NAA), and under different light conditions, in the Department of Ornamental Plants of the Faculty of Biotechnology and Horticulture of the Agricultural University in Kraków (Poland). The following types of lighting with an intensity of 60 μmol m^−2^ s^−1^ were applied to each hormonal treatment: monochromatic red light (647–770 nm, Philips TLD, 36 W lamp), blue light (450–492 nm, Philips TLD, 36 W lamp) and UV-A (360–450 nm, Philips TLD 36 W lamp). Illumination with white light (390–760 nm, Tungsram F33, 40 W lamp) was adopted as the control conditions with the same hormonal treatments. Throughout the entire cultivation cycle (6 weeks), a 12/12 h photoperiod was applied. In addition, cultures were performed in permanent darkness for 6 weeks. Subsequently, the biomass was harvested and dried in fresh air at room temperature. The biomass increment was calculated by dividing sample dry weight by the dry weight of inoculum.

### HPLC Analysis

Dried plant material was milled and extracted with 50 ml of boiling methanol (analytical grade) for 3 h at 78 ± 2 °C. After filtering the samples, the methanol was evaporated to dryness and the remains were dissolved in 4 ml of methanol (HPLC grade). Methanolic extracts from the cultured biomass and the commercial herbal preparation were analysed by HPLC. A liquid chromatograph (Merck-Hitachi) was used with a DAD detector; the column was Purospher® RP-18e (250 × 4 mm, 5 μm) (Merck-Hitachi), gradient elution with methanol: 0.5% acetic acid and 20:80 to 100:0% (*v*/*v*) at 25 ± 2 °C. Quantitative determinations were carried out at a wavelength of *λ* = 254 nm (spectrum recorded in the range *λ* = 200–400 nm) [26]. Compounds were identified based on retention times and UV spectra.

The reference standards used for HPLC analyses were specific for skullcaps flavonoids: baicalein^a^, baicalin^b^, chrysin^a^, scutellarein^a^, scutellarin^b^, wogonin^b^, wogonoside^a^; other flavonoids: apigenin^a^, apigetrin^e^, cynaroside^a^, hyperoside ^a^, isorhamnetin 3-rhamnoside^e^, kaempferol^c^, kaempferol 3-rhamnoside^e^, kaempferol 7-rhamnoside^e^, quercetin^a^, quercimetrin^e^, quercitrin^a^, luteolin^e^, myricetin^a^, populnin^e^, rhamnetin^e^, rutoside^a^, trifolin^e^, vitexin^a^; phenolic acids: chlorogenic^a^, 3,4-dihydroxyphenylacetic^a^, ferulic^a^, gallic^a^, gentisic^a^, hydrocaffeic^a^, *p*-hydroxybenzoic^a^, isoferulic^a^, caffeic^a^, *m*-coumaric^a^, *o*-coumaric^a^, *p*-coumaric^a^, neochlorogenic^a^, protocatechuic^a^, rosmarinic^a^, salicylic^a^, sinapic^a^, syringic^a^, vanillic^a^; their precursors: benzoic^d^ and cinnamic acids^a^; and also phenylethanoid glycosides: verbascoside^b^, isoverbascoside^b^; (a—Sigma-Aldrich), (b—ChromaDex), (c—Roth), (d—Merck), (e—isolated in the Department of Pharmacognosy, Medical University of Gdańsk, Poland).

### Plant Material

A commercially available herbal preparation of *S. lateriflora* (from NANGA Company, Poland) was used for comparison purposes.

### Statistical Analysis

The obtained results are expressed as the mean ± standard deviation of four independent determinations. Statistical significance of differences was evaluated using a one-way ANOVA followed by post hoc test comparisons (Tukey’s HSD test). The differences were statistically significant when *p* < 0.05. The analyses were conducted using Statistica ver.12.5 (StatSoft, Poland).

## Results

### Biomass Production

**Fig. 1:**
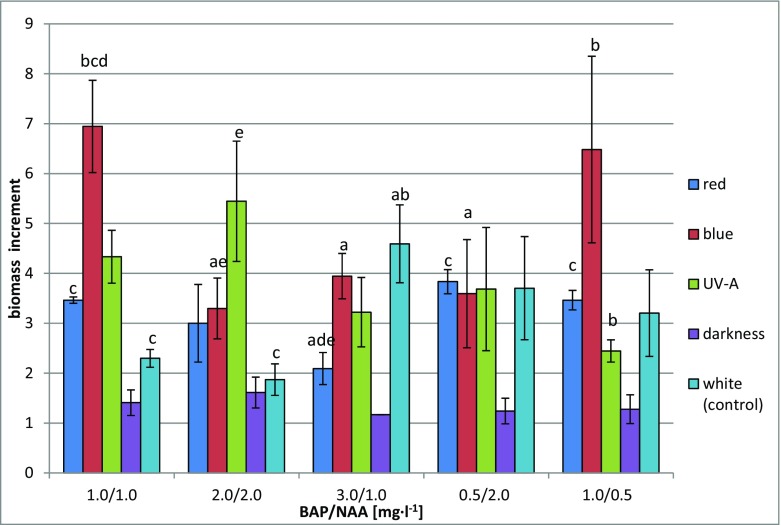
Increments of *S. lateriflora* L. in vitro cultures dry biomass grown on MS medium variants under different hormonal combinations and light conditions after 6 weeks of culture. Statistically significant differences *p* < 0.05: *a*—vs. MS variant 1.0/1.0; *b*—vs. MS variant 2.0/2.0; *c*—vs. MS variant 3.0/1.0; *d*—vs. MS variant 0.5/2.0; *e*—vs. MS variant 1.0/0.5

The increases in dry biomass of shoots during the 6-week growth cycle ranged from 1.17- to 6.94-fold (Fig. 1). The medium on which the largest increase in biomass was recorded was the MS variant containing 1 mg l^−1^ BAP and 1 mg l^−1^ NAA (especially under blue light). The medium containing 1 mg l^−1^ BAP and 0.5 mg l^−1^ NAA was also favourable to increases in biomass (max. 6.48-fold). A much greater influence on the growth of biomass was exerted by the lighting conditions applied. The largest increase in biomass was found in cultures grown under blue light. Smaller maximum increases were obtained under white light (4.6-fold) and UV-A irradiation (5.44-fold). The least favourable conditions proved to be the absence of light. The increases in the biomass of shoots grown in the dark were the smallest (max. 1.61-fold) (Fig. 1).

### Accumulation of Flavonoids

**Fig. 2:**
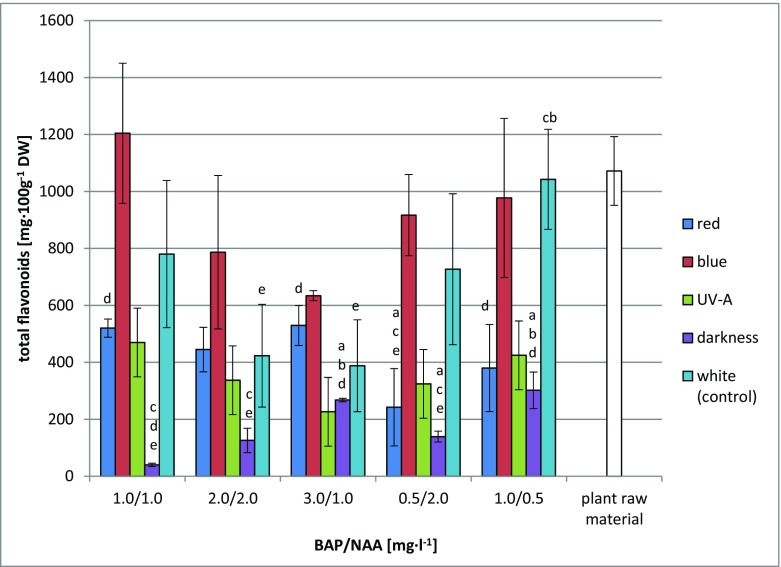
Total content of flavonoids [mg 100 g^−1^ DW ± SD] in methanolic extracts from biomass of *S. lateriflora* L. in vitro cultures grown on MS medium variants under different hormonal combinations and light conditions after 6 weeks of culture. Statistically significant differences *p* < 0.05: *a*—vs. MS variant 1.0/1.0; *b*—vs. MS variant 2.0/2.0; *c*—vs. MS variant 3.0/1.0; *d*—vs. MS variant 0.5/2.0; *e*—vs. MS variant 1.0/0.5

The methanolic extracts tested for 26 compounds were found to contain four flavonoids specific to the genus *Scutellaria*: baicalein, baicalin, wogonin and wogonoside. None of the flavonoids popular in the world of plants had its presence confirmed. The largest accumulated amounts were those of baicalin (max. 886.44 mg 100 g^−1^ dry weight (DW)). Wogonoside was also accumulated in considerable quantities (max. 293.84 mg 100 g^−1^ DW). The total flavonoid content of the biomass varied markedly (39.90–1204.33 mg 100 g^−1^ DW). The highest total flavonoid content was found on two variants of MS medium containing, respectively, 1 mg l^−1^ BAP and 1 mg l^−1^ NAA (1204.33 mg 100 g^−1^ DW) and 1 mg l^−1^ BAP and 0.5 mg l^−1^ NAA (1042.73 mg 100 g^−1^ DW). The most favourable lighting conditions for the accumulation of flavonoids were provided by blue light. Depending on the MS variant, the total flavonoid content under blue light varied between 634.09 and 1204.33 mg 100 g^−1^ DW. This was from 0.94 to 1.86 times higher than under white light. The secondary metabolites were accumulated in the smallest quantities in the dark and in the presence of UV-A radiation. Detailed results are shown in Fig. 2.

The quantitatively dominant metabolites were baicalin and wogonoside, glucuronides of baicalein and wogonin. The highest baicalin content (886.44 mg 100 g^−1^ DW) was found in extracts from the biomass grown on the MS variant containing 1 mg l^−1^ BAP and 1 mg l^−1^ NAA (Table 1). This medium was the best under all the illumination conditions tested, except for white light and darkness. The accumulation of baicalin was promoted by blue light, 0.96–2.00 times higher than under white light, whereas the least favourable was the absence of light, 13.37 mg 100 g^−1^ DW (Table 1). The variant of MS medium containing 1 mg l^−1^ BAP and 1 mg l^−1^ NAA favourable to the accumulation of baicalin was also the best production medium for wogonoside (60.63–293.84 mg 100 g^−1^ DW), 1.32–2.06 times higher than under white light. Blue light also proved to be the most favourable for the accumulation of this glycoside. In the absence of light, wogonoside was not accumulated (Table 1). Baicalein and wogonin were accumulated in much lower quantities. Their maximum levels did not exceed 95 and 50 mg 100 g^−1^ DW, respectively. The presence of baicalein was not confirmed in the biomass grown under blue light and UV-A irradiation (Table 1).

**Table 1:** The amounts of estimated metabolites in extracts from biomass of *S. lateriflora* cultured in vitro on MS medium variants under different hormonal combinations and light conditions after 6 weeks of culture [mg 100 g^-1^ DW ± SD] (− indicates not detected)

Light conditions	Medium variant BAP/NAA [mg l^−1^]	Estimated metabolites [mg 100 g^−1^ DW]					
		Baicalein	Wogonin	Baicalin	Wogonoside	3,4-Dihydroxy-phenylacetic acid	Verbascoside
Red	1.0/1.0	7.29 ± 0.62	24.12 ± 3.37***	428.05 ± 22.11	60.63 ± 6.01	22.03 ± 1.91	123.52 ± 11.13^#,##^
	2.0/2.0	4.88 ± 1.43	21.82 ± 3.21***	367.76 ± 51.43	50.35 ± 5.18	12.01 ± 1.89	75.48 ± 0.00
	3.0/1.0	7.70 ± 1.24	49.42 ± 11.54*^,^**^,#,##^	420.31 ± 57.72	51.90 ± 6.63	30.71 ± 6.65	128.97 ± 31.63^#,##^
	0.5/2.0	7.09 ± 1.16	16.04 ± 1.40***	192.98 ± 89.33	26.23 ± 13.58	12.48 ± 2.84	57.00 ± 13.79*^,^***
	1.0/0.5	5.91 ± 1.13	22.49 ± 6.79***	303.25 ± 89.50	48.29 ± 13.40	29.85 ± 9.20	60.49 ± 15.02*^,^***
Blue	1.0/1.0	–	24.06 ± 5.57	886.44 ± 127.79***	293.84 ± 53.34	16.08 ± 2.54	125.67 ± 18.5
	2.0/2.0	–	32.82 ± 5.06	635.34 ± 138.40	118.42 ± 21.94	24.95 ± 4.37	93.31 ± 28.27
	3.0/1.0	–	25.93 ± 1.60	491.65 ± 13.80*	116.50 ± 6.72	11.49 ± 3.05	54.51 ± 6.08
	0.5/2.0	–	39.57 ± 12.25	704.52 ± 100.05	172.93 ± 15.56	24.45 ± 7.85	169.15 ± 62.45
	1.0/0.5	–	28.56 ± 11.49	761.08 ± 101.87	187.55 ± 92.91	10.66 ± 3.23	108.15 ± 15.64
UV-A	1.0/1.0	–	14.89 ± 2.35	395.65 ± 109.64	59.20 ± 16.79	17.85 ± 2.76	64.41 ± 11.44
	2.0/2.0	–	14.70 ± 6.00	266.26 ± 17.04	56.10 ± 16.81	17.78 ± 4.47	110.00 ± 27.98***^,#,##^
	3.0/1.0	–	18.37 ± 6.10	169.57 ± 22.35	38.35 ± 4.38	11.77 ± 4.89	47.96 ± 16.44**
	0.5/2.0	–	14.33 ± 5.70	263.75 ± 93.99	46.26 ± 14.81	21.81 ± 7.98	51.04 ± 3.86**
	1.0/0.5	–	15.11 ± 1.29	358.83 ± 112.35	50.68 ± 14.01	18.92 ± 5.67	40.23 ± 8.68**
Darkness	1.0/1.0	5.68 ± 1.04***	20.85 ± 2.03***	13.37 ± 2.51**^,^***^,#,##^	–	–	14.68 ± 17.22***^,#,##^
	2.0/2.0	3.59 ± 1.12***^,##^	20.29 ± 6.51***	101.86 ± 24.50*^,^***^,##^	–	–	41.77 ± 9.80
	3.0/1.0	28.31 ± 5.47*^,^**^,#,##^	33.36 ± 1.25*^,^**	205.95 ± 0.48*^,^**^,#^	–	–	109.41 ± 0.26*
	0.5/2.0	8.33 ± 0.78***	27.31 ± 3.24	103.57 ± 10.70*^,^***^,##^	–	–	111.39 ± 14.64*
	1.0/0.5	14.31 ± 2.10**^,^***	26.80 ± 1.53	260.37 ± 46.13*^,^**^,#^	–	–	122.94 ± 41.26*
White (control)	1.0/1.0	15.24 ± 1.94	29.33 ± 5.45	592.63 ± 158.17	142.98 ± 45.28	33.56 ± 4.8***	88.67 ± 19.14
	2.0/2.0	10.04 ± 4.00	33.47 ± 7.45	317.92 ± 106.0	61.79 ± 31.95	19.49 ± 2.70	100.64 ± 18.89
	3.0/1.0	8.19 ± 3.10	14.53 ± 6.00	306.64 ± 96.78	58.66 ± 20.10	6.07 ± 2.27*	51.31 ± 24.20
	0.5/2.0	94.82 ± 43.38	40.09 ± 13.13	484.50 ± 157.19	107.51 ± 26.01	24.43 ± 8.68	130.20 ± 42.57
	1.0/0.5	84.21 ± 33.01	24.70 ± 6.32	792.17 ± 136.49	141.65 ± 18.85	18.54 ± 4.49	70.29 ± 12.91

Statistically significant differences: *p < 0.05 vs. MS variant 1.0/1.0; **p < 0.05 vs. MS variant 2.0/2.0; ***p < 0.05 vs. MS variant 3.0/1.0; ^#^ *p* < 0.05 vs. MS variant 0.5/2.0; ^##^ *p* < 0.05 vs. MS variant 1.0/0.5

The total flavonoid content of the commercial herbal preparation was 1071.85 mg 100 g^−1^ DW (Fig. 1). The material was found to contain the same compounds as the in vitro cultures and additionally scutellarin. The predominant metabolites were scutellarin and wogonoside, in the amounts of 511.32 and 434.09 mg 100 g^−1^ DW, respectively.

### Accumulation of Other Phenolic Compounds

HPLC analyses for 19 phenolic acids and also benzoic acid and cinnamic acid found the presence of only 3,4-dihydroxyphenylacetic acid. The analyses of methanolic extracts also revealed the presence of other phenolic compound: a phenylethanoid glycoside–verbascoside. None of the samples analysed were found to contain its isomer–isoverbascoside.

The highest concentration of 3,4-dihydroxyphenylacetic acid (33.56 mg 100 g^−1^ DW) was found in the presence of white light, on the medium containing 1 mg l^−1^ BAP and 1 mg l^−1^ NAA, but it was not accumulated in the dark (Table 1).

The verbascoside content in the biomass extracts ranged from 14.68 to 169.15 mg 100 g^−1^ DW. The best production medium for verbascoside was the MS variant containing 0.5 mg l^−1^ BAP and 2 mg l^−1^ NAA. As for the accumulation of flavonoids, blue light proved to be the most favourable to the accumulation of this metabolite (0.93–1.54 times higher than under white light). The least favourable conditions to the accumulation of verbascoside in analysed shoot cultures were irradiation with UV-A and the absence of light (Table 1).

The most favourable lighting conditions that can be nominated for the accumulation of all analysed secondary metabolites in shoot cultures of *S. lateriflora* are those provided by blue light. The best productive medium proved to be the variant of MS medium containing 1 mg l^−1^ BAP and 1 mg l^−1^ NAA.

Analysis of the commercial herbal preparation found the presence of protocatechuic acid, caffeic acid and the depsides—chlorogenic and rosmarinic acids and verbascoside. The verbascoside content was particularly high (715.72 mg 100 g^−1^ DW).

## Discussion

Both the concentrations of plant growth regulators and light conditions tested in our study had an effect on biomass growth (Fig. 1). The increases in biomass were moderate, except in the dark (1.17–1.61-fold), and good in the case of blue light (6.5–6.9-fold), especially on the MS variants containing 1 mg l^−1^ BAP and 1 mg l^−1^ NAA and 1 mg l^−1^ BAP and 0.5 mg l^−1^ NAA. Under the control conditions (white light), the biomass increased 1.9–4.6-fold (Fig. 1). Blue light had also contributed the most to biomass growth in the case of *Ruta graveolens* shoot cultures and shoot-differentiated callus cultures of *R. graveolens* ssp. *divaricata* [27], as well as in shoot-differentiated callus cultures of *Schisandra chinensis* [28].

The biomass from *S. lateriflora* cultures was found to contain baicalein, baicalin, wogonin and wogonoside. The presence of other flavonoids specific to the genus *Scutellaria* was not confirmed. The dominant metabolite was baicalin (max. 886.44 mg 100 g^−1^ DW). The accumulated amounts of wogonoside were smaller than those of baicalin (max. 293.84 mg 100 g^−1^ DW). Both compounds are glucuronides of baicalein and wogonin, respectively, which are evidence of a high capacity of *S. lateriflora* cultures in vitro for biosynthesis and accumulation of glycosidic conjugates. Baicalin is also the main metabolite estimated by us in in vitro cultures of another medicinal species of the genus *Scutellaria*—*S. baicalensis*. In studies by other research centres, the dominant flavonoids in *S. lateriflora* cultures in vitro have been the aglycones, baicalein and wogonin [19].

The highest total amounts of flavonoids we found were in the biomass grown on those variants of MS medium that also promoted increases in biomass, i.e. 1 mg l^−1^ BAP and 1 mg l^−1^ NAA and 1 mg l^−1^ BAP and 0.5 mg l^−1^ NAA—also good for the accumulation of verbascoside. The most favourable to the accumulation of flavonoids were the conditions that were also favourable to biomass growth, i.e. those provided by blue light (0.94 to 1.86 times higher than under white light). By contrast, the lowest capacity for the accumulation of flavonoids was demonstrated in the dark (0.05 to 0.69 times greater than under white light).

The accumulation of verbascoside in the tested cultures was confirmed under all the experimental conditions. The maximum amounts of verbascoside in the biomass grown under different light conditions did not vary much (110.0–169.1 mg 100 g^−1^ DW). They were considerably (4.7–6.5 times) lower than in shoot cultures of *S. baicalensis* grown in our laboratory. The highest verbascoside content was found on the MS medium containing 0.5 mg l^−1^ BAP and 2 mg l^−1^ NAA under blue light. Analyses of *S. lateriflora* callus cultures performed by other team had determined verbascoside as the main metabolite, with no difference in the accumulation of this compound in the presence or absence of white light [19]. Our team had also investigated the influence of white light and its absence on the accumulation of verbascoside in callus cultures of *Verbena officinalis*. In that model, the composition of the medium and the amounts of growth regulators were found to have a greater effect on the accumulation of verbascoside than the type of lighting, although the verbascoside content was higher in the presence of light [29]. Despite reports in the literature on the presence of phenolic acids in the aerial parts of *S. lateriflora*, our study confirmed only the presence of 3,4-dihydroxyphenylacetic acid. This compound has proven antiproliferative and apoptotic properties [30].

Our analysis of flavonoids in the commercially available herbal preparation revealed that the total amount of this group of compounds in the herb was similar to that in the biomass from in vitro cultures (1.07 and 1.20 g 100 g^−1^ DW). However, the qualitative composition and the percentages of individual flavonoids were markedly different. In both cases, the dominant compounds were flavonoid glycosides. Also, in the case of phenolic acids, the compounds whose presence was confirmed in the herb were not present in the biomass in vitro.

In cultures with a high degree of differentiation of *S. lateriflora*, as well as of other plant species, large amounts of secondary metabolites, such as coumarins, lignans, phenolic acids and flavonoids, can be obtained [31]. A significant effect of the addition of various concentrations of plant growth regulators to the medium on the accumulation of metabolites in shoot cultures in vitro has been proven by us for species such as *R. graveolens* [32, 33], *S. chinensis* [34, 35], *Aronia melanocarpa* [36], and *Hypericum perforatum* cultivars [37, 38]. Also, for *S. lateriflora*, this effect is significant, and we can nominate the MS variant containing 1 mg l^−1^ BAP and 1 mg l^−1^ NAA as the best production medium.

The influence of light on the growth and development of cultures in vitro has long been known and studied. Light is also one of the environmental factors that can affect the accumulation of secondary metabolites, which is particularly important in the case of medicinal plants. For *S. lateriflora* cultures, the effect of light on the accumulation of flavonoids proved to be greater than the effect of the composition of the culture medium. Also, in the case of verbascoside, this effect was evident [29]. By far, the most significant effect was demonstrated by the use of blue light. We had demonstrated similar effects in our studies of different groups of secondary metabolites in cultures of *R. graveolens*, *R. graveolens* ssp*. divaricata* [27], *S. chinensis* [28], *A. melanocarpa, A. arbutifolia* and *Aronia* × *prunifolia* [39]. In the case of the accumulation of phenolic acids in *R. graveolens* and *R. graveolens* ssp*. divaricata*, comparatively good lighting conditions proved to be those provided by white light. In the case of furanocoumarins, the results were varied. In *R. graveolens* cultures, furanocoumarins were best accumulated under blue light, whereas in *R. graveolens* ssp*. divaricata* cultures, in the dark. The small increases in the biomass of *S. lateriflora* cultures in darkness may be due to the fact that they are shoot cultures. In vitro cultures of non-differentiated tissues (callus, orchid protocorms, fern gametophytes) can be grown in darkness, but with increasing degree of organogenesis, the need for light increases [40]. The cultures of *S. lateriflora* grown in darkness also accumulated the least amounts of secondary metabolites. A similar relationship has been observed in *Cyclopia subternata* callus cultures, where darkness reduced the accumulation of most bioflavonoids tested [41]. By contrast, in callus cultures of *Ammi majus*, darkness clearly stimulated the accumulation of bergapten [42].

This means that each time, empirical selection of in vitro culture conditions is necessary. Blue light also stimulates the accumulation of isoflavones and hesperidin in *C. subternata* cultures [41], of flavonoids in *Alternanthera* sp. cultures [43] and of anthocyanins in *Haplopappus gracilis* and *Populus* sp. cultures [44]. This type of lighting also stimulates the accumulation of phenolic compounds and increases the antioxidant capacity of in vitro cultures of *Rehmannia glutinosa* [45]. There are various theories explaining the stimulating effect of illumination with blue light on the accumulation of metabolites. This effect may be a result of interactions both on the genomic level and the protein level associated with the evolution of chloroplasts and the functioning of photosynthesis [46, 47].

## Conclusions

Our study proved a significant effect of the concentration of plant growth regulators in the culture medium on the accumulation of specific for *Scutellaria* genus flavonoids and verbascoside in shoot cultures of *S. lateriflora* L. It also demonstrated a significant influence of lighting conditions (monochromatic light, white light and absence of light) on the accumulation of secondary metabolites. The main compounds proved to be baicalin and verbascoside. The best medium promoting the growth of biomass and the production and accumulation of flavonoids proved to be the variant of MS medium containing 1 mg l^−1^ BAP and 1 mg l^−1^ NAA, and the highest amounts of the compounds analysed were obtained in the presence of blue light. In vitro cultures of *S. lateriflora* L. may constitute a potential source for obtaining biologically active secondary metabolites, as an alternative to the raw material of natural origin. In vitro cultures are performed under laboratory conditions, under which a change in the physical conditions, including lighting, makes it possible to optimize the accumulation of the compounds produced by the cultures.
